# Study of chronic orofacial pain with preclinical models

**DOI:** 10.1186/1744-8069-10-S1-O6

**Published:** 2014-12-15

**Authors:** Alaa ABD-Elsayed, Kevin Kohan, Xiaozhuo Zuo, Meuonghoon Cha, Jennifer Ling, Jianguo Gu

**Affiliations:** 1 Department of Anesthesiology, College of Medicine, University of Cincinnati, Cincinnati, OH 45267, USA

## Background

Chronic orofacial pain conditions such as trigeminal neuropathic pain, reflex sympathetic dystrophy of the face, and temporomandibular pain disorders are debilitating pain conditions. They are known to respond poorly to treatment including opioids in human patients. There is a pressing need to better understand the mechanisms of these chronic orofacial pain conditions so that new and effective therapeutic targets can be identify. To achieve this goal, preclinical animal models that well represent human chronic orofacial pain conditions are needed. In the present study we used two preclinical models of chronic orofacial pain, the chronic constriction nerve injury of the infraorbital nerve (ION-CCI model) and the oxaliplatin-induced orofacial pain (oxaliplatin model). We applied newly developed orofacial operant behavioral assessment to study orofacial pain and to test effects on Kv7.2 channel activators in alleviating orofacial pain in these animals.

## Materials and methods

Male Sprague–Dawley rats (300–450 g) were used in this study. In the ION-CCI model a chronic constriction nerve injury was created using unilateral ligation of the infraorbital nerve. For oxoliplatin model of orofacial pain, oxaliplatin was administered to rats (*i.p.*) at 2 mg/kg per day for five consecutive days. Orofacial pain behaviors in responses to mechanical and cold stimuli were assessed by the orofacial operant behavioral assessment method. To test the effects of Kv7.2 activators in alleviating orofacial pain in these two preclinical models, retigabine or CF341 was administered to these animals at the doses ranging from 0.19 to 15 mg.

## Results

Rats of both ION-CCI model and oxaliplatin model showed significant orofacial mechanical allodynia and cold allodynia/hyperalgesia as examined by using the orofacial operant assessment method. Retigabine, a classical Kv7.2 channel activator, significantly alleviated orofacial cold allodynia in both ION-CCI model and oxaliplatin model**.** CF341, a newly synthesized Kv7.2 channel activator, also showed alleviation of the orofacial cold allodynia with efficacy similar to retigabine.

## Conclusions

In both ION-CCI model and oxaliplatin model, orofacial operant behavioral assessment gives quantitative measurements of chronic orofacial pain and therapeutic effects of Kv7.2 activators. The preclinical models shown in this work are useful for both mechanistic study and drug development for chronic orofacial pain.

